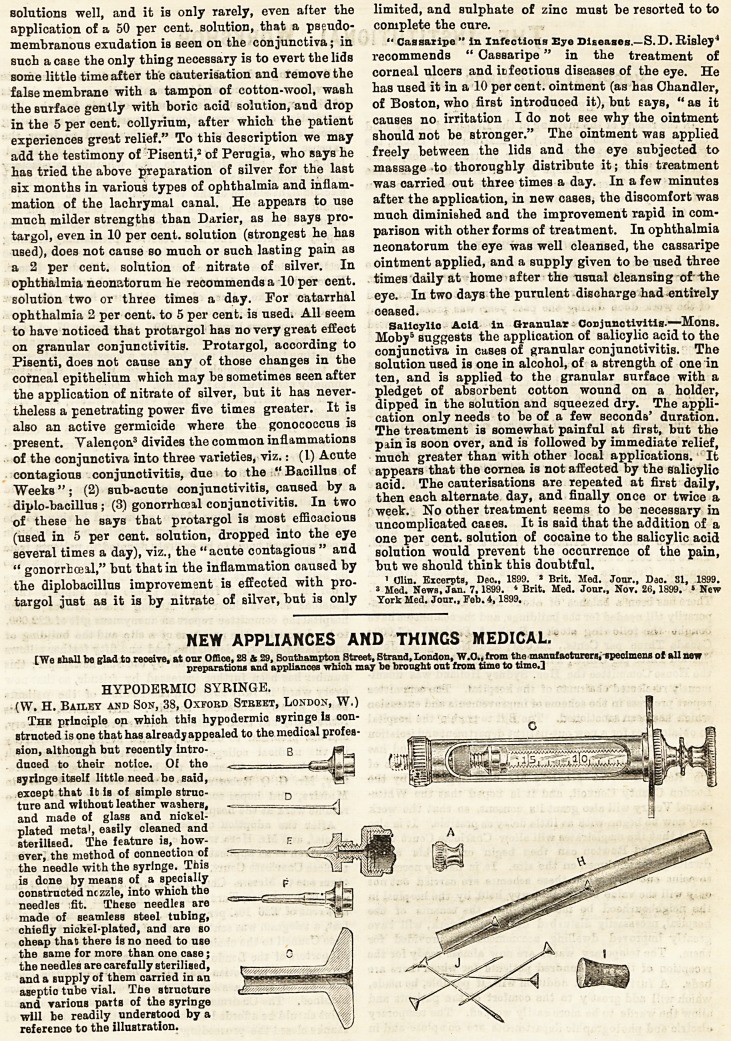# New Appliances and Things Medical

**Published:** 1899-03-11

**Authors:** 


					NEW APPLIANCES AND THINGS MEDICAL.
[We "hill be glad to receive, at onr Office, 28 & 29, Southampton Street, Strand, London, W.O., from the manufacturers, specimens of all new
preparations and appliances which may be brought out from time to time.]
HYPODERMIC SYRINGE.
(W. H. Bailey and Son, 38, Oxford Stbeet, London, W.)
The principle on which thfs hypodermic syringe ia con-
structed is one that has already appealed to the medical profes-
Bion, although bat recently intro-
duced to their notice. Of the
By ringe itself little need be said,
except that ib is of simple struc-
ture and without leather washers,
and made of glass and nickel-
plated metal, easily cleaned and
sterilised. The feature is, how-
ever, the method of connection of
the needle with the syringe. This
is done by means of a specially
constructed nczzle, into which the
needles fit. These needles are
made of seamless steel tubing,
chiefly nickel-plated, and are so
cheap that: there is no need to use
the same for more than one case;
the needles are carefully sterilised,
and a Bupply of them carried in an
aseptio tube vial. The structure
and various parts of the syringe
will be readily understood by a
reference to the illustration.
solutions well, and it is only rarely, even after the limited, and sulphate of zinc must be resorted to to
application of a 50 per cent, solution, that a pseudo- complete the cure.
membranous exudation is seen on the conjunctiva; in " Cassaripe " In infections Eye Diseases.?S.D. Risley4
such a case the only thing necessary is to evert the lids recommends "Cassaripe" in the treatment of
some little time after the cauterisation and remove the corneal ulcers and infectious diseases of the eye. He
false membrane with a tampon of cotton-wool, wash has used it in a 10 per cent, ointment (as has Chandler,
the surface gently with boric acid solution, and drop of Boston, who first introduced it), but says, " as it
in the 5 per cent, collyrium, after which the patient causes no irritation I do not see why the ointment
experiences great relief." To this description we may should not be stronger." The ointment was applied
add the testimony of Pisenti,2 of Perugia, who says he freely between the lids and the eye subjected to
has tried the above preparation of silver for the last massage to thoroughly distribute it; this treatment
six months in various types of ophthalmia and infiam- was carried out three times a day. In a few minutes
mation of the lachrymal canal. He appears to use after the application, in new cases, the discomfort was
much milder strengths than Darier, as he says pro- much diminished and the improvement rapid in com-
targol, even in 10 per cent, solution (strongest he has parison with other forms of treatment. In ophthalmia
uBed), does not cause so much or such lasting pain as neonatorum the eye was well cleansed, the cassaripe
a 2 per cent, solution of nitrate of silver. In ointment applied, and a supply given to be used three
ophthalmia neonatorum he recommends a 10 per cent, times daily at home after the usual cleansing of'iihe
solution two or three times a day. For catarrhal eye. In two days the purulent discharge had-entirely
ophthalmia 2 per cent, to 5 per cent, is used. All seem ceased.
to have noticed that protargol has no very great effect Salicylic Acid in Granular Conjunctivitis.?Mons.
on granular conjunctivitis. Protargol, according to Mol?S "gSSestB the application of salicylic acid to the
t,. , 'J . ,, ? , conjunctiva in cases of granular conjunctivitis. The
Pisenti, does not cause any of those changes in the soluJtion used is one in alcohol> of a 8tJrengfch of one in
corneal epithelium which may be sometimes Been after ten, and is applied to the granular surface with a
the application of nitrate of silver, but it has never- pledget of absorbent cotton wound on a holder,
theless a penetrating power five times greater. It is dipped in the solution and squeezed dry. The appli-
also an active germicide where the gonococcus is ??ljr nfe?s to be of a few seconds' duration.
i. -rr 1 o J- -J o -a The treatment is somewhat painful at first, but the
present. Yalen^on divides the common inflammations pajn js soon over> an(j ?s followed by immediate relief,
of the conjunctiva into three varieties, viz.: (I) Acute much greater than with other local applications. It
contagious conjunctivitis, due to the " Bacillus of appears that the cornea is not affected by the salicylic
Weeks"; (2) sub-acute conjunctivitis, caused by a acid. The cauterisations are repeated at first daily,
diplo-bacillus; (3) gonorrhceal conjunctivitis. In two then each alternate day, and finally once or twice a
of these he says that protargol is most efficacious
, , . c , ,. , -j ? j ._ uncomplicated cases. It is said that the addition of a
(used m 5 per cent, solution, dropped into the eye one per cent solution of cocaine to the saiicylic acid
several times a day), viz., the "acute contagious " and solution would prevent the occurrence of the pain,
" gonorrhceal," but that in the inflammation caused by but we should think this doubtful.
the diplobacillus improvement is effected with pro- 1 Win* Excerpts, Deo., 1899. * Brit. Med. Jour., Dao. 31, 1899.
targol just as it is by nitrate of silver, but is only Y^kfd.'jourtri'eb!^1i899.Bnt* Med" Jonr" Nov* 26' 1899* ' New
NEW APPLIANCES AND THINGS MEDICAL.
[We shall be glad to receive, at onr Office, 28 & 29, Southampton Street, Strand, London, W.O., from the manufacturers, specimens of all new
preparations and appliances whioh may be brought out from time to time.]
HYPODERMIC SYRINGE. .... .
(W. H. Bailey and Son, 38, Oxford Street, London, W.)
The principle on which thfs hypodermic syringe ia con-
structed is one that has already appealed to the medical profes-
sion, although but recently intro-
duced to their notice. Of the
syringe itself little need be said,
except that it is of simple struc-
ture and without leather washers,
and made of glass and nickel-
plated metaJ, easily cleaned and
sterillBed. The feature is, how-
ever, the method of connection of
the needle with the syringe. This
is done by means of a specially
constructed nczzle, into which the
needles fit. These needles are
made of seamless steel tubing,
chiefly nickel-plated, and are so
cheap that there is no need to use
the same for more than one case;
the needles are carefully sterilised,
and a supply of them carried in an
aseptio tube vial. The structure
and various parts of the syringe
will be readily understood by a
reference to the illustration.

				

## Figures and Tables

**Figure f1:**